# Bridging Worlds: Can Evidence, Regulation and Indigenous Leadership Shape the Future of Traditional, Complementary and Integrative Medicine?

**DOI:** 10.1002/hpm.70139

**Published:** 2026-09-12

**Authors:** Abdul‐Basit Abdul‐Samed, Dina Balabanova, Neil Spicer, Martin McKee

**Affiliations:** ^1^ Faculty of Public Health and Policy London School of Hygiene & Tropical Medicine London UK

**Keywords:** health systems, Indigenous knowledge, integration, misinformation, regulation, traditional medicine

## Abstract

**Background and need:**

As the WHO Global Traditional Medicine Strategy 2025–2034 encourages countries to consider integrating traditional, complementary and integrative medicine (TCIM) into health systems, there is a need to clarify the principal scientific, regulatory, ethical, and governance challenges involved. Renewed global attention to TCIM, including at the Second Global Summit on Traditional Medicine held in New Delhi in December 2025, has intensified calls for approaches that are safe, effective, equitable, and culturally respectful.

**Objective:**

This commentary examines the principal challenges that must be addressed if TCIM is to be integrated safely and equitably into health systems, with particular attention to evidence generation, regulation, commercialisation, misinformation, Indigenous knowledge governance, and models of health‐system integration.

**Methods:**

We undertook a policy‐oriented narrative review informed by an interpretive reading of the WHO Global Traditional Medicine Strategy 2025–2034 and related governance frameworks, including the Convention on Biological Diversity and the Nagoya Protocol. We then conducted iterative, non‐systematic searches across major bibliographic databases and policy sources to identify literature and examples relevant to five core domains shaping TCIM integration: evidence and economics, regulation, commercial exploitation, misinformation, and Indigenous knowledge.

**Main findings and conclusion:**

Integrating TCIM into health systems requires more than evidence of clinical effectiveness alone. Progress depends on methodological approaches capable of assessing complex and context‐dependent interventions; stronger regulatory and pharmacovigilance systems; safeguards against misleading commercial claims and digital misinformation; and, crucially, Indigenous leadership in the governance, protection, and co‐production of knowledge, so that benefit‐sharing and research priority‐setting rest on community authority rather than consultation alone. Integration should be understood as a spectrum, from coexistence and referral to co‐location and fully blended models, with implementation shaped by local health‐system capacity and patterns of real‐world use. Advancing TCIM will therefore require coordinated action grounded in evidence, ethics, equity, and culturally respectful governance.

## Highlights

- Evidence, ethics and equity are essential for safe integration of traditional, complementary and integrative medicine (TCIM).
- TCIM integration is a multi‐domain governance challenge, not only a question of clinical efficacy.
- Plural, fit‐for‐purpose evidence frameworks are needed rather than a narrow “more trials” agenda.
- Indigenous leadership is central to ethical integration, evidence co‐production and fair benefit‐sharing.
- Integration is a spectrum that can itself create the conditions for better research and accountability.

## 1 Introduction

In late 2025, the WHO and the Government of India convened the Second Global Summit on Traditional Medicine in New Delhi [1]. This gathering marked a pivotal moment in the international effort to define the role of traditional, complementary and integrative medicine (TCIM) in modern health systems, given the rapidly growing interest in this area. The summit brought together ministers, scientists, Indigenous leaders and practitioners from more than 100 countries, underscoring the global momentum behind a more integrated and inclusive approach to healthcare. WHO Director‐General Dr Tedros Adhanom Ghebreyesus emphasised that TCIM could help address the major health threats of the modern world, particularly inequities in access to care, and stressed that “traditional wisdom and modern science are not inconsistent, but complementary to each other” [2].

TCIM encompasses a diverse array of practices, products, and knowledge systems. These include herbal medicine and plant‐derived therapies; spiritual healing and faith‐based interventions; acupuncture, bone‐setting, and massage; and community‐embedded roles such as traditional birth attendants [3]. Among the most globally recognised formal systems are traditional Chinese medicine (TCM), grounded in concepts of yin‐yang balance and the five elements: wood (木), fire (火), earth (土), metal (金) and water (水), and Ayurveda, originating from the Indian subcontinent, which frames health as equilibrium among three fundamental energies (doshas: Vata, Pitta, and Kapha) [4, 5]. In West Africa, healing traditions are embedded within cosmological frameworks specific to different ethnocultural groups: Akan healing practices in Ghana integrate herbalism with spiritual diagnostics that relate illness to problems with relationships between the living and the ancestral world [6], while Mande healing traditions across Mali, Guinea, and neighbouring countries similarly situate the healer as an intermediary between social and spiritual realms. Unani medicine, rooted in Graeco‐Arabic scholarship, remains practised across parts of South Asia, the Middle East and North Africa [7]. This plurality of practices and cosmologies is the defining feature of the global TCIM landscape and the primary reason why no single governance architecture seems to suffice. TCIM should therefore not be treated as a homogeneous intervention category: products, practices, and knowledge systems differ in standardisation, mechanisms, ownership, risks, evidence requirements, and regulation, and evidence from one modality should not be generalised to TCIM as a whole.

The summit aligned its agenda with the WHO Global Traditional Medicine Strategy 2025–2034, adopted at the 78th World Health Assembly in May 2025 [3]. The Strategy outlines a framework focused on evidence, safety, equity and culturally respectful integration of TCIM into national health systems. It emphasises evidence‐based practice, stating that no TCIM intervention should be recommended without rigorous scientific evaluation, while acknowledging the need for diverse research methods suitable for complex, holistic practices. It also sets out a regulatory architecture for products, practitioners, and service delivery to ensure quality, trust, and risk‐based oversight, and underscores the protection of Indigenous knowledge, benefit sharing, and biodiversity as essential to responsible global practice.

The WHO's vision is not to replace conventional care but to integrate safe, effective, evidence‐based TCIM into primary healthcare and broader policy where appropriate. Integration is a spectrum, with service‐delivery models ranging from basic mutual recognition between biomedical and traditional practitioners to co‐location, referral networks, and fully blended pathways delivered by dual‐trained providers. At the governance level, it includes regulatory recognition, quality standards and accountability. For the workforce, it requires curricular reform, accreditation and defined scopes of practice. Financing decisions hinge on which TCIM interventions are evidence‐based and warrant public reimbursement. By strengthening research capacity and fostering respectful cross‐cultural partnerships, the WHO aims to rebalance relationships among people, communities and the planet. The New Delhi Summit represents a key step, but the process ahead remains complex and demanding [8]. Throughout this paper, we use “integration” broadly to refer to a spectrum of relationships between TCIM and formal health systems, ranging from coexistence and structured referral to co‐location and fully blended clinical pathways; it does not imply a single model or the co‐option of TCIM into biomedical institutions [9, 10]. We likewise use “Indigenous leadership” to mean leadership exercised by Indigenous peoples and communities over the governance, interpretation, and application of their own knowledge systems, including research, regulation, and benefit‐sharing; this should be distinguished from leadership within codified traditional medicine systems such as Ayurveda, TCM, or Unani, which may have long‐standing institutional authorities but different historical foundations and are not necessarily Indigenous in the same sense (Box 1). This focus reflects Indigenous peoples' distinctive collective authority over their knowledge, biological resources, research priorities, data, and benefit‐sharing, often in contexts shaped by colonisation, dispossession, and appropriation; it does not diminish the importance of leadership within codified systems.

**Box 1 Definitions and concepts**

1 **Traditional, complementary and integrative medicine (TCIM):** A plural category encompassing codified traditional medical systems, Indigenous and community‐based healing traditions, herbal and plant‐derived therapies, spiritual healing, manual therapies, and other practices operating alongside or within formal health systems. “Traditional knowledge” is used as the broader term, while “Indigenous knowledge” refers specifically to knowledge held and governed by Indigenous peoples and communities.

**Integration:** A spectrum of relationships between TCIM and formal health systems, ranging from coexistence and referral to co‐location and fully blended care pathways; it does not imply a single model or automatic co‐option by biomedical institutions.

**Indigenous leadership:** Leadership by Indigenous peoples and communities over the governance, protection, interpretation, and application of their own knowledge systems, including research, regulation, and benefit‐sharing.

**Evidence spectrum:** A plural and context‐sensitive understanding of evidence that includes, but is not limited to, trials; it also encompasses pragmatic, observational, implementation, mixed‐methods, and participatory approaches suited to complex TCIM interventions and settings.

While many governments and practitioners have welcomed the WHO Global Traditional Medicine Strategy, it has also prompted debate within parts of the scientific and medical community. Critics have cautioned that efforts to institutionalise TCIM may risk appearing to endorse interventions whose evidence base remains limited, or whose biological plausibility is disputed, if rigorous standards of evidence are not consistently applied [11, 12, 13]. Others have raised concerns about potential tensions between culturally embedded practices and scientific evaluation [14], arguing that WHO must balance inclusivity with a clear commitment to evidence‐based practice. These debates do not diminish the value of the Strategy, but they do point to a deeper question that a narrow framing can obscure: integration requires more than applying conventional biomedical hierarchies of evidence to plural knowledge systems. A growing literature on pluralistic health systems and the decolonisation of health science argues that rigid evidence hierarchies can systematically devalue knowledge generated outside randomised designs and instead calls for context‐sensitive, methodologically inclusive frameworks capable of accommodating traditional knowledge [12, 13]. Our aim in this commentary is therefore not simply to reiterate that “more evidence is needed”, but to examine the challenges that must be addressed, across evidence, regulation, commerce, communication, and Indigenous governance, if TCIM is to be integrated safely and equitably into health systems.

## 2 Methods

This paper is a policy‐oriented narrative review. Our objective was not to catalogue the clinical evidence for individual TCIM modalities exhaustively, but to synthesise heterogeneous forms of evidence, clinical, economic, legal, regulatory, governance, and implementation‐related, across domains that are conceptually linked but methodologically diverse. We deliberately chose a narrative approach because the questions raised by TCIM integration span epistemically distinct bodies of knowledge that no single systematic design could accommodate; this choice sacrifices systematic exhaustiveness within any one domain in exchange for a cross‐cutting policy analysis, and we indicate throughout where focused systematic reviews would add value.

To identify the organising domains, we undertook an interpretive policy analysis of the WHO Global Traditional Medicine Strategy 2025–2034 [3] and related governance instruments, including the Convention on Biological Diversity [15] and the Nagoya Protocol on Access and Benefit‐Sharing [16]. Rather than a formal, coding‐based qualitative content analysis, we read these instruments interpretively to surface the recurring policy concerns they foreground, and organised our commentary around five interlinked domains: evidence and economics, regulation, commercial exploitation, misinformation, and Indigenous knowledge and leadership (Table 1). Consistent with this framing, we treat “robust evidence” pluralistically throughout, encompassing not only conventional randomised designs where appropriate but also mixed‐methods, pragmatic, implementation, and comparative‐effectiveness studies, as well as evidence frameworks capable of incorporating traditional knowledge and context‐sensitive practice [12, 13].

**TABLE 1:** Five domains shaping TCIM integration.

Domain	Principal risks	Opportunities	Policy levers
Evidence and economics	Weak or uneven evidence on clinical effectiveness, safety, and cost‐effectiveness may lead to either the premature endorsement of ineffective interventions or the blanket dismissal of potentially valuable ones; narrow methodological expectations may also fail to capture complex, individualised, or culturally embedded practices.	Fit‐for‐purpose evaluation can identify TCIM interventions that are safe, effective, affordable, and relevant to unmet needs, while broadening the evidence base to include pragmatic, implementation, mixed‐methods, and comparative‐effectiveness approaches suited to real‐world practice.	Invest in research capacity; support plural evidence frameworks; strengthen pharmacovigilance and health technology assessment; commission economic evaluations; and develop shared standards for data collection and outcome reporting.
Regulation	Fragmented or under‐resourced regulation can permit poor‐quality products, contamination, misleading labelling, weak practitioner oversight, and cross‐border regulatory leakage through online sales; over‐burdensome regulation can also exclude smaller producers and drive practice underground.	Strong, risk‐based regulation can improve product quality, practitioner accountability, and public trust, while regional harmonisation can reduce fragmentation and support safer trade and integration.	Build national regulatory capacity; strengthen laboratory, inspection, and pharmacovigilance systems; develop public practitioner/product registries; support regional harmonisation; and align oversight with risk rather than one‐size‐fits‐all rules.
Commercial exploitation	Commercialisation may encourage unsupported therapeutic claims, inflated prices, inequitable access, and consumer financial harm, especially where products are aggressively marketed without clear evidence, where export demand displaces local use, or where unsustainable harvesting causes biodiversity loss, ecosystem damage, and harms to local livelihoods.	Appropriate market governance can improve transparency, reduce predatory practices, and support access to safe, affordable products while allowing innovation, responsible commercial development, and fair and equitable benefit‐sharing with knowledge‐holding communities.	Restrict unsupported health claims; require disclosure of evidence and transparent labelling; strengthen consumer protection law; monitor pricing and market concentration; require access and benefit‐sharing arrangements where relevant; integrate biodiversity and sustainable‐harvesting safeguards; and ensure that public reimbursement decisions are evidence‐informed.
Misinformation	Unverified claims about TCIM can spread rapidly online, delay appropriate care, encourage unsafe self‐medication, and deepen distrust in public health institutions; misinformation may arise from lay therapeutic traditions, commercial marketing, or politically motivated attacks on scientific governance.	Better communication can improve public understanding, support informed use of TCIM, and reduce harmful polarisation between community knowledge and biomedical advice. Community co‐designed messaging may also be more persuasive than top‐down fact‐checking alone.	Develop culturally grounded health communication; strengthen health literacy and digital literacy; collaborate with platforms to flag harmful claims; regulate AI‐enabled impersonation and false endorsements; and protect the independence of scientific and public health institutions.
Indigenous knowledge and leadership	Traditional knowledge may be appropriated, commercialised, or documented without consent or fair return; weak legal protection, unequal bargaining power, and externally imposed research agendas can undermine community authority and trust.	Indigenous leadership can strengthen ethical governance, improve the legitimacy of evidence generation, protect community knowledge systems, and support fair benefit‐sharing, culturally grounded research, and sustainable practice.	Embed prior informed consent and mutually agreed terms; use access and benefit‐sharing frameworks; recognise Indigenous data and intellectual sovereignty; support community‐led governance and research; and align conservation, industrial, and health policies around sustainability and equity.

We conducted iterative searches between January 2024 and February 2026 across MEDLINE, Embase, Web of Science, Scopus, and JSTOR, using terms related to *traditional medicine*, *integrative medicine*, *regulation*, *evidence*, *misinformation*, and *Indigenous knowledge*. These dates refer to when the searches were performed, not to a restriction on publication dates: we prioritised recent literature where possible, but also included older foundational studies, regulatory precedents, and conceptual works where these remained directly relevant to the policy issues under discussion. Alongside clinical and safety studies, we deliberately sought health systems strengthening and policy analysis literature, legal and regulatory materials, and governance frameworks, reflecting the multi‐domain nature of the questions. We included English‐language peer‐reviewed studies, systematic reviews, legal and policy documents, and selected commentaries. Contextual factors such as regulatory capacity, cultural norms, and health‐system structure informed our interpretation, as did our own expertise in global health policy and health systems. We stress that this paper is intended to inform the agenda for future work, which could involve more systematic reviews of specific issues. It is therefore not designed to provide exhaustive evidence grading across TCIM modalities, formulations, or health‐system models, but to identify cross‐cutting governance challenges that should guide more focused future reviews and evaluations.

## 3 Building the Evidence Base and Economic Case for TCIM

### 3.1 Establishing Clinical Effectiveness

Several methodological challenges remain in establishing the clinical effectiveness of many TCIMs, particularly those that are highly individualised or combine faith and pharmacology. Several herbal monotherapies, such as standardised extracts of *Hypericum perforatum* (St John's wort) [17] or Allium sativum (garlic) [18], have well‐characterised phytochemical profiles and pharmacological pathways, illustrating that clear mechanisms can be established when formulations are consistent and amenable to laboratory investigation. This has, however, proven challenging for manufacturers of some products, such as the Ayurvedic formulations *Trikatu* and Ashwagandha, which have demonstrated potential biological efficacy, but achieving consistent quality of materials for formal evaluation has been difficult [19, 20]. Multi‐component herbal preparations and highly individualised modalities often pose particular difficulties for evaluation and reproducibility, though this challenge is not uniform across all TCIM traditions. Established regulatory systems, such as those governing TCM in China or Ayurveda in India, maintain codified diagnostic frameworks, standardised pharmacopoeias, and quality specifications that enable more systematic evaluation. The reproducibility challenges documented in the literature apply mostly to practices that are highly individualised, operate outside formal regulatory oversight, or rely on practitioner‐specific formulations derived from esoteric or orally transmitted knowledge in which treatment composition, dose, and application can vary substantially between practitioners and even between consultations [19, 21]. Acupuncture is among the more extensively studied TCIM practices, and large individual‐patient data meta‐analyses demonstrate small‐to‐moderate effects for chronic pain [22], although many trials have major methodological limitations [23]. In general, however, these findings remain the subject of ongoing scientific debate, particularly regarding the contributions of contextual and placebo‐related factors, as well as the clinical significance of the reported effect sizes. These examples are illustrative rather than exhaustive; a comprehensive assessment of individual formulations would require modality‐specific systematic reviews.

Some interventions with origins in TCIM have well‐established empirical foundations. These include artemisinin‐based therapies (derived from and long used in TCM), which are now the WHO‐recommended first‐line treatment for malaria, representing perhaps the most consequential validation of a traditional medicine intervention in modern history [24]. Mindfulness‐based cognitive therapy (MBCT) has demonstrated a 31% reduction in depressive relapse risk in individual patient data meta‐analyses, and yoga shows moderate reductions in depressive symptoms, comparable in effect size to antidepressant medication [25, 26]. Some Cochrane‐reviewed evidence demonstrates symptom resolution with extracts for acute respiratory infections (although the evidence is of low quality) [27]. That said, such interventions represent a minority: many others would require innovative study designs to accommodate complexity, variation in practitioner expertise, and culturally embedded therapeutic logics [28]. Recent methodological work highlights how the personalisation intrinsic to many TCIM interventions strains conventional parallel‐group trial designs, and motivates adaptive, n‐of‐1, pragmatic, and mixed‐methods alternatives better suited to context‐dependent practice [29]. However, this creates an obvious problem for regulators. Are they being asked to approve the intervention or the individual administering it (or even the combination of the skills and characteristics of the practitioner and the belief system of the patient)? This question is rarely asked.

Clinical evaluation must, moreover, consider both safety and efficacy. For many TCIM products and practices, adverse effects may be underreported due to fragmented pharmacovigilance systems, inconsistent product standardisation, weak practitioner registration, and the widespread but mistaken assumption that “natural” implies safety. Safety assessment must therefore extend beyond reported side effects in trials to encompass contamination, adulteration, herb–drug interactions, and the possibility that harms remain unrecognised in routine use—concerns we return to in the discussion of regulation and pharmacovigilance below.

### 3.2 Moving From Effectiveness to Cost‐Effectiveness

Across sub‐Saharan Africa, traditional medicine is frequently the first and most accessible recourse for illness. Affordability, flexible payment arrangements and geographic proximity are consistently among the strongest pull factors towards traditional medicine use in these settings [30]. Crucially, this cost advantage is not just absolute price: even when traditional practitioners charge fees that appear substantial, they commonly accept deferred payment, payment in kind, or payment contingent on successful treatment, financing arrangements that biomedical facilities rarely accommodate. When the full costs of accessing biomedical care are properly accounted for (consultation fees, prescribed medicines, laboratory investigations, transport to a distant facility, and lost wages), the financial advantage of biomedical services frequently disappears, particularly for households in the lowest income quintiles [31, 32]. In Ghana, for instance, 42% of the poorest insured patients continued to incur significant out‐of‐pocket costs despite enrolment in the National Health Insurance Scheme [33]. Traditional medicine, therefore, can function as a low‐cost option. This reality must inform both the economic case for TCIM integration and the design of financing policies.

If TCIMs have been shown to be safe and effective, it is important to conduct economic evaluations to determine their viability at scale. This introduces additional complexities related to cost structures, labour models, and health system integration. Economic studies of acupuncture, for instance, have identified it as cost‐effective for chronic low back pain in several high‐income settings [34] although cost‐effectiveness results are highly context‐dependent: labour costs, practitioner density, patterns of insurance coverage, and regulatory requirements vary significantly between health systems.

Economic evaluations should consider not just the cost of ingredients but also the cost of the entire treatment package. Some traditional herbal medicines are inexpensive but labour‐intensive to administer, while others have become more costly due to globalised supply chains and shifts in market demand [35, 36]. This heterogeneity highlights the need for sophisticated economic modelling that accounts for variation in product costs, practitioners' remuneration, regulatory compliance burdens [21], and the extent of TCIM integration into existing service‐delivery platforms [37]. Such modelling is essential for informing policy, assessing market opportunities, and identifying sustainable pathways to adoption [38]. A full‐cost perspective should also account for biodiversity loss, depletion of medicinal species, ecosystem damage, cultivation and restoration costs, supply‐chain sustainability, and effects on local livelihoods. However, few governments have the capacity to undertake such comprehensive assessments.

Framing the task solely as one of generating more evidence, however, risks obscuring a prior and more contested question: which forms of evidence count as legitimate, in what contexts, and under whose authority. Recent work on evidence‐informed decision‐making in TCIM suggests that the central difficulty is less an absolute shortage of studies than the absence of agreed, pluralistic frameworks for weighing heterogeneous evidence, including traditional knowledge, in guideline and policy development [13]. These judgements are rarely neutral; where they rest on an unexamined biomedical hierarchy, they can foreclose precisely the knowledge systems that integration is meant to respect. A recurring theme in this literature, and one to which we return below, is that Indigenous and community stakeholders should help set research priorities and validate evidence, rather than being consulted only after evidentiary standards have already been fixed [12, 39, 40]. This does not imply abandoning fundamental scientific principles of transparency, reproducibility, critical appraisal, and protection from harm; rather, it means applying them through methods and governance arrangements appropriate to the intervention, context, and knowledge system being evaluated.

### 3.3 Purchasing Decisions and Coverage Pathways

Establishing cost‐effectiveness is only a first step in deciding whether to include any treatment in a benefit package. Regardless of how health systems are financed, authorities considering the inclusion of TCIM must decide which of their scarce resources to allocate to it. Countries that have attempted this illustrate the political and methodological challenges involved. Germany's decision to delist homoeopathic remedies from statutory health insurance was, in part, a consequence of scientific evaluation concluding that homoeopathy lacked robust proof of efficacy. However, it also reflected prolonged political and professional debate, including pressure from medical associations advocating for evidence‐based reimbursement, concerns about the symbolic legitimacy conferred by public funding, and tensions between federal and regional health authorities [41]. These dynamics illustrate that purchasing decisions are often shaped not only by scientific evidence but also by broader institutional, political, and professional considerations that influence how evidence is interpreted and acted upon. South Korea's retention of separate benefit schedules for Korean medicine [42] reflects a mix of professional lobbying, public demand, and institutional path dependencies. These experiences demonstrate how purchasing decisions must weigh multiple factors: the strength of the evidence, societal preferences, cultural legitimacy, and budgetary resources.

These upstream supply chain dynamics interact closely with the broader regulatory and enforcement challenges that govern TCIM products and practitioners at the point of delivery, which we examine next.

### 3.4 Regulating TCIM Products and Practices

Regulating TCIM products and practices presents significant challenges, largely due to the diversity of treatments, the variability of national regulatory capacities, and the difficulty of establishing uniform standards across jurisdictions. Countries differ substantially in their ability to develop and enforce robust regulatory frameworks. Importantly, these challenges operate at two distinct levels: within national borders, where governments must oversee domestic production and practice; and across borders, where the rapid growth of international trade and online purchasing means that consumers can readily access TCIM products that have been manufactured, marketed, and sold outside any single national regulatory jurisdiction.

The experience of the European Union's Traditional Herbal Medicinal Products Directive (THMPD) illustrates the challenges involved in attempting to standardise requirements for quality, safety, and evidence. Evaluations of the Directive's implementation report that smaller manufacturers struggled to meet Good Manufacturing Practice requirements, raising concerns about reduced market diversity, although the desirability of this remains contested [43, 44]. China's longstanding regulation of traditional Chinese medicine (TCM) demonstrates that robust frameworks, reinforced by Good Agricultural and Collection Practices (GACP) and detailed guidance, can improve product quality, but this requires substantial investment in laboratory capacity, inspection systems, and sustained state investment [21]. In contrast, evidence from South Asia and Latin America shows that implementing risk‐based regulation, that is, oversight based on potential harm, is difficult without sustained government investment and adequately resourced regulatory and inspection bodies [35, 45].

The second challenge arises from the fact that many TCM products are now exported worldwide. The challenge is not confined to countries with weaker domestic oversight: the growth of e‐commerce means that consumers everywhere can access products sold outside national regulatory systems. At the same time, some countries with more limited regulatory capacity may derive safety benefits from importing products manufactured under stronger Good Manufacturing Practice and labelling regimes elsewhere. While such trade falls under the provisions of, for example, the World Trade Organisation (WTO) Sanitary and Phytosanitary Agreement and its Technical Barriers to Trade Agreement, at least for the 164 WTO member states, these agreements depend on adequate enforcement capacity, which also affects domestic trade. Thus, studies of herbal medicine markets in sub‐Saharan Africa have documented substantial variability in product quality, contamination with heavy metals or pharmaceuticals, and inconsistent labelling, reflecting differences in regulatory capacity, market structures, and the scale of informal practice [46]. Ghana's regulatory landscape is shaped by relatively well‐established institutions such as the Food and Drugs Authority and the Traditional Medicine Practice Council [47], whereas Nigeria's experience reflects the difficulties of extending oversight to a far larger and more decentralised herbal market [48]. Recognising this heterogeneity is important: while regional patterns can be observed, the specific regulatory gaps and priorities vary by country and require tailored approaches rather than broad generalisations.

Despite these challenges, several promising solutions demonstrate how regulatory systems can be strengthened. One approach is to build the capacity of national regulatory authorities. The African Medicines Regulatory Harmonisation programme illustrates how regional cooperation can enhance regulatory effectiveness by pooling expertise, harmonising standards, and accelerating joint product assessments across multiple countries [49]. Improved surveillance systems also contribute significantly to product safety. For example, the introduction of systematic pharmacovigilance structures for Ayurvedic, Unani, and Siddha medicines in India has increased the detection of previously rare adverse events, thereby enabling more effective risk management [50].

Regional harmonisation efforts further support regulatory alignment and reduce fragmentation. The Association of Southeast Asian Nations (ASEAN) has advanced common standards for TCIMs, including joint technical documents on safety and quality assessment, demonstrating how coordinated frameworks can promote trade while elevating regulatory standards [51]. At the global level, the WHO has attempted to strengthen coherence by expanding the use of tools such as the Global Benchmarking Tool, which assesses the maturity of national regulatory authorities, and by providing targeted capacity‐building programmes to support improvements in laboratory capacity, inspection skills, and practitioner oversight [52]. Increasing transparency, such as through public registries of licenced practitioners and approved products, is also increasingly recognised as essential to building public trust and enabling safe integration of TCIM into health systems. Yet transparency measures and registration requirements alone are insufficient if the incentive structures facing practitioners are not addressed. Many countries have only informal or no systems for accrediting practitioners. Even where they exist, evidence from across sub‐Saharan Africa and South Asia shows that many traditional medicine practitioners are reluctant to register, disclose knowledge, or participate in formal integration processes [53]. This resistance is rarely irrational: it frequently reflects legitimate fears about the appropriation of intellectual property, loss of practice autonomy, competitive disadvantage in formally regulated markets, and the accumulated experience of having traditional knowledge documented, commercialised, or discredited without acknowledgement or benefit to the originating community. In some settings, maintaining secrecy of medicinal knowledge is the only effective protection against such outcomes [54]. Integration strategies that rely on disclosure and registration without addressing these underlying concerns risk generating superficial compliance while further driving substantive knowledge‐sharing underground. Sustained engagement, demonstrable respect for practitioners' expertise, and concrete benefit‐sharing arrangements built on the prior‐informed consent principles already embedded in the Nagoya Protocol are prerequisites to building trust.

### 3.5 Preventing Commercial Exploitation

Preventing commercial exploitation in increasingly globalised TCIM markets is a critical challenge, particularly in private sector‐dominated settings where profit incentives may overshadow safety and evidence. Three major risks stand out: misleading therapeutic claims, inequitable pricing, and inadequate consumer financial protection.

A major risk arises from inaccurate or exaggerated marketing claims unsupported by scientific evidence. Analyses of the U.S. dietary supplement industry have documented widespread promotional statements lacking clinical substantiation, which contribute to inappropriate consumer expectations and, in some cases, delay timely access to effective biomedical care [55, 56]. Similar patterns have been observed in Asia, where expanding markets for products such as Kampo in Japan have been accompanied by aggressive marketing strategies. A second form of exploitation emerges through inequitable pricing. Research on Ayurvedic and herbal products in South Asia shows that prices often rise sharply after commercialisation or entry into export markets. These increases can limit access for local populations and generate tensions between commercial demand and community use [36]. The third major risk involves insufficient consumer financial protection. Studies from Europe and Australia show that TCIM users frequently underestimate long‐term costs, partly due to unclear pricing structures and optimistic marketing claims. Strengthening consumer protection is therefore essential, particularly as TCIM markets expand and become more commercially sophisticated.

Several regulatory approaches offer promising models. Australia has legislated a risk‐based framework under which sponsors must hold evidence supporting listed‐medicine indications, with the TGA conducting post‐market compliance reviews and publishing outcomes for selected medicines [57]. However, because listed medicines are generally entered on the Australian Register of Therapeutic Goods through sponsor self‐certification and only selected products undergo post‐market review, the published findings do not provide a comprehensive estimate of market‐wide compliance [58]. Comparable disclosure is not routine in many other national regulatory systems. Although such labels may help consumers distinguish between traditional‐use claims and scientifically substantiated indications, publicly available compliance data remain limited, and it is unclear how well consumers understand the distinction. The European Union, by contrast, restricts the use of health claims through the Nutrition and Health Claims Regulation, limiting promotional statements to those supported by approved evidence. These models illustrate how regulatory tools can help curtail misleading marketing, promote transparency, and reduce consumer vulnerability by combining disclosure requirements with restrictions on unsupported claims.

### 3.6 Addressing Misinformation and Public Communication

It is essential to address the growing risk of misinformation about TCIM, especially online, where unverified claims can circulate rapidly and pose significant public health risks. Studies have shown that misinformation about herbal remedies and “natural cures” spreads widely on social media, often outperforming accurate health information in terms of reach and engagement [59, 60]. During the COVID‐19 pandemic, false claims about traditional remedies such as neem, ginger, or herbal vapour therapies circulated widely, contributing to delays in seeking care and inappropriate self‐medication [61].

It is important to understand the reasons for this happening. The first involves therapeutic claims that circulate within communities and are grounded in longstanding lay knowledges, practices, and accumulated lived experience, but have not been subjected to formal scientific testing. Such practices often reflect generations of empirical observation; the challenge is not that this knowledge is inherently unreliable, but that in the absence of systematic evaluation, it is difficult to distinguish remedies that are genuinely effective from those that are ineffective or even harmful. The second is commercially motivated misinformation, in which manufacturers or sellers make unsupported claims to drive sales. This category has taken on new dimensions with the emergence of AI‐generated content: fake social media profiles and automated bots impersonating health professionals are now used to lend credibility to unsubstantiated product claims, making it increasingly difficult for consumers to distinguish legitimate clinical advice from marketing. The third involves the use of health misinformation for political purposes, that is, situations in which public figures or political movements promote scepticism towards conventional medicine and scientific institutions to advance ideological or electoral agendas. Recent events in the United States, where senior political figures have publicly rejected established scientific evidence on vaccines and pharmaceutical regulation, illustrate how holders of institutional authority can amplify distrust and weaken the regulatory systems on which public health depends [62]. Each of these problems requires distinct policy responses, such as community‐based health communication that respects and builds upon traditional knowledge, advertising regulation, platform‐level controls on AI‐generated impersonation, and institutional safeguards that protect the independence of scientific governance from political interference.

TCIM misinformation also has a distinctive structural dimension. Because traditional knowledge systems have historically been marginalised within biomedical hierarchies, what scholars of epistemic injustice term a systematic devaluation of non‐Western ways of knowing, communities may respond to perceived dismissal by retreating further from conventional health advice. This dynamic can paradoxically fuel the very misinformation that public health agencies seek to counter. Culturally aligned narratives are more persuasive and harder to counter through conventional fact‐checking alone [62], suggesting that effective TCIM communication strategies must be co‐designed with communities and build upon, rather than dismiss, existing knowledge frameworks.

Although the WHO strategy emphasises the need to generate evidence and support public understanding, concrete mechanisms for regulating harmful digital content remain limited. Public health agencies and governments will therefore need to strengthen algorithmic safeguards, collaborate with platforms to flag false claims, and invest in high‐quality, multilingual health communication. Evidence also indicates that strengthening health literacy and digital literacy, alongside community‐based communication through trusted local intermediaries, can reduce susceptibility to health misinformation [63].

### 3.7 Protecting Indigenous Knowledge and Ensuring Ethical Governance

A fifth consideration is how to protect the property rights associated with Indigenous knowledge while promoting scientific research, recognising that significant power asymmetries exist. International legal instruments highlight both the opportunities and challenges in this space. The Convention on Biological Diversity [15] and the Nagoya Protocol on Access and Benefit‐Sharing [16] provide the clearest global frameworks that affirm Indigenous peoples' sovereign rights over biological resources and associated traditional knowledge, and require prior informed consent and mutually agreed terms before research or commercial use. Empirical analyses show that Access and Benefit‐Sharing mechanisms can strengthen community control when implemented effectively, as evidenced by benefit‐sharing agreements with the San peoples of Southern Africa regarding the Hoodia plant [64]. However, uneven national implementation and limited community legal capacity frequently undermine these protections.

Legal scholarship also emphasises that intellectual property regulations often fail to safeguard collective, intergenerational knowledge, thereby enabling misappropriation, as evidenced by the well‐documented patents filed on neem, turmeric, and *Ayahuasca* without community consent [65]. In response, instruments such as the World Intellectual Property Organisation's (WIPO) Intergovernmental Committee's draft texts on traditional knowledge propose culturally grounded intellectual property models that extend beyond standard patent law, including community‐held rights and defensive publication systems [66]. Much of the relevant literature relates to intellectual property associated with foodstuffs, such as Ethiopian coffee [67].

To avoid commodification while supporting ethical research, evidence points to the need for binding ethical research protocols grounded in Indigenous governance, such as the First Nations Ownership, Control, Access, and Possession principles in Canada [68], and the Aotearoa/New Zealand guidelines for Māori data sovereignty [69]. Community‐owned knowledge repositories demonstrate how documentation initiatives can protect against ‘biopiracy’ while preserving autonomy [70]. Such mechanisms are essential to ensuring that traditional knowledge is respected, safeguarded, and used responsibly. Ultimately, the ethical use of Indigenous knowledge and resources depends on governance models designed with, rather than for, communities, ensuring that research, benefit‐sharing, and decision‐making remain grounded in Indigenous authority, autonomy, and consent.

Crucially, Indigenous leadership extends beyond protecting knowledge from misappropriation to shaping how evidence itself is produced and validated. Where Indigenous researchers and communities lead the design of studies, setting the questions, defining the outcomes that matter, and determining which methods are culturally appropriate, evidence generation becomes both more legitimate and, on its own terms, more rigorous. Reviews of Australian policy on First Peoples' cultural medicines, for example, show how Indigenous‐led methodologies such as yarning can validate knowledge while meeting standards of analytical transparency, and how the absence of such leadership has historically produced policies that misrepresent or erase community knowledge [40]. Positioning Indigenous peoples as co‐producers of evidence frameworks, rather than as subjects of research designed elsewhere, is therefore integral to, not separate from, the evidentiary and regulatory tasks discussed earlier.

Sustainability is a related and growing concern as biodiversity loss accelerates. Evidence of overharvesting species such as *Prunus africana* and *Nardostachys jatamansi* illustrates how poorly regulated global demand can undermine ecosystems and local livelihoods [21, 36]. Export regulations, such as India's restrictions on *Rauvolfia serpentina*, further illustrate how governments attempt to balance commercial opportunity with conservation and equity considerations [38]. These dynamics demonstrate that industrial and environmental policies are integral to both the long‐term economic viability and the ecological and cultural sustainability of TCIM practices.

### 3.8 Integrating TCIM Into Health Systems

Drawing on existing health‐services frameworks, integration of safe, effective, and cost‐effective TCIM into formal health services can be viewed as a spectrum of intensities across the health system (Figure 1) [9, 10, 71, 72]. The suitability of any model depends on the specific modality, system design, provider capacity, and regulatory alignment; evidence from one modality or setting should not be generalised to TCIM as a whole.

**FIGURE 1:**
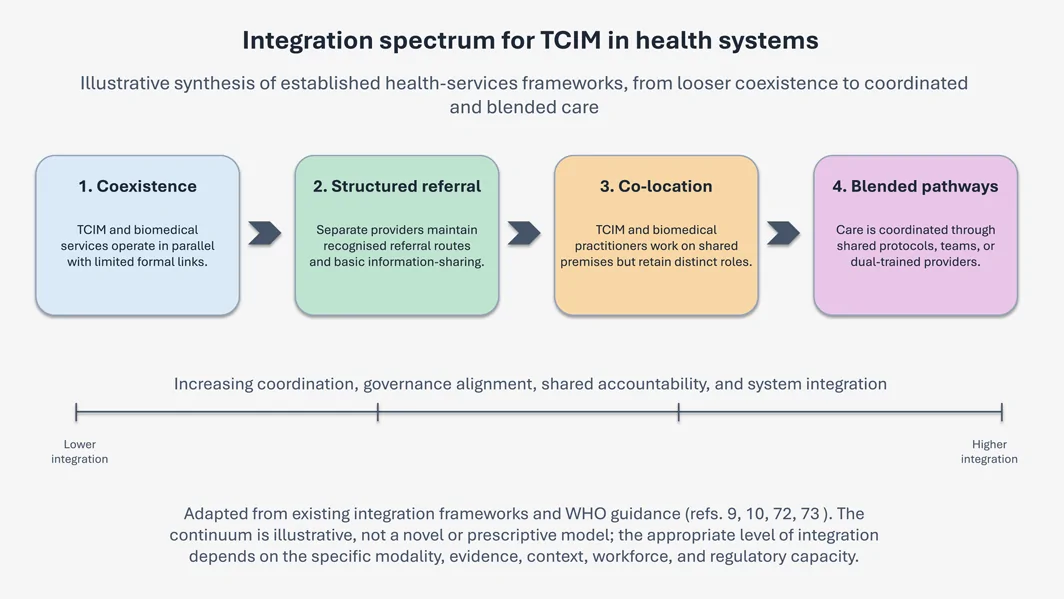
Illustrative progression from looser coexistence to more coordinated and blended models of care. The figure shows integration as a spectrum, beginning with parallel operation of TCIM and biomedical services, moving through structured referral and co‐location, and culminating in blended pathways involving shared protocols, coordinated teams, or dual‐trained providers.

At its simplest, integration begins with mutual awareness and information‐sharing: primary care providers knowing which TCIM services operate locally, what they offer, and engaging with practitioners and patients who use them. This step requires no structural change but can improve the quality of care by helping clinicians account for concurrent TCIM use in clinical decision‐making and by reducing mistrust between biomedical and traditional providers.

A further step is co‐location, in which TCIM practitioners work alongside biomedical providers on shared premises while maintaining distinct scopes of practice. Ghana offers one of the most formalised examples in Africa. Since a Ministry of Health pilot in 2011, university‐trained medical herbalists (holders of a Bachelor of Science in Herbal Medicine) have been placed in public hospital outpatient departments under a shared governance structure [73]. By the mid‐2020s, around 55 such sites operated nationwide, with traditional medicine consultations partially reimbursed through the National Health Insurance Scheme. However, herbal products remain outside insurance coverage, limiting the depth of integration achievable for patients.

India provides further examples where biomedical and Unani or Ayurvedic practitioners are co‐located in public primary care facilities, enabling patients to access both systems during a single visit [36]. Co‐location can improve coordination and reduce fragmentation, but it depends on clear role delineation and shared protocols to avoid duplication or conflict [71, 72].

A middle model is the use of referral networks, in which biomedical and TCIM providers work independently while maintaining structured pathways for patient referrals. Evaluations of Brazil's Integrative and Complementary Practices Programme show that these models work best when services are decentralised and community‐driven, thereby enabling access to TCIM while preserving provider specialisation [74].

At the highest integration level are fully blended clinical pathways, where dual‐trained providers or coordinated teams deliver TCIM and biomedical care as a unified model. China exemplifies this approach: many primary care practitioners are trained in both TCM and biomedicine and draw on both in practice, though concerns persist about quality variation and tensions between diagnostic paradigms [71].

Korea's dual‐licenced physician system offers a similarly formalised model with promise in chronic disease management, but it requires extensive training and strong regulatory alignment [75]. These approaches also carry major workforce implications, as scaling dual‐trained providers demands substantial curricular reform, deeper integration of knowledge systems, and sustained institutional investment. Such models need not, however, be limited to physicians. In many settings, there is scope for dual‐licenced or upskilled nurses, midwives, pharmacists, herbal dispensers, psychologists, physiotherapists, and other allied health practitioners to play important bridging roles between TCIM and biomedical care, particularly where community roles and professional boundaries already overlap. This matters not only for herbal and pharmacological modalities but also for the non‐pharmacological and self‐care dimensions of TCIM, manual therapies, movement practices, and community‐based support, which biomedical services are often not designed to deliver.

Across all integration models, patient perspectives and demand‐side dynamics warrant greater attention. Research consistently shows that patients frequently navigate between biomedical and traditional systems concurrently, often without disclosing this to either provider [76]. Understanding these patterns of medical pluralism, including the out‐of‐pocket cost implications and decision‐making processes that drive them, is essential for designing integration models that reflect how people actually seek care, rather than how health systems assume they should. Integration also offers an opportunity to strengthen dimensions of care that biomedical models are not designed to deliver, such as community self‐reliance and health creation, which some have described as a “fourth tier” of the health system, centred on individuals' and communities' own capacity for self‐care [77].

The relationship between evidence and integration is, moreover, not one‐way. In some settings, partial integration into formal health services can itself create a positive feedback loop: by improving case ascertainment, standardising practice, and generating the institutional platforms, patient volumes, referral pathways, and regulatory visibility needed for research, integration can help make better evidence and evaluation possible rather than simply waiting on them. The trajectories of TCM in China, Korean medicine in South Korea, and medical herbalism in Ghana each suggest that structured integration and evidence generation can advance together [9, 10].

To support cross‐country learning, the WHO could lead the development of a comparative evidence framework and facilitate pilot evaluations. Research consistently highlights the importance of standardising data collection through shared classifications, minimum datasets, and common outcome indicators to ensure comparability across diverse contexts. The inclusion of a chapter on Traditional Medicine in the 11th revision of the International Classification of Diseases demonstrates the feasibility of establishing global standards and enables more systematic monitoring of utilisation and outcomes [78]. Importantly, this also provides a foundation for evaluating real‐world implementation across different integration models, allowing countries to compare performance, safety, and patient‐reported outcomes within and between systems. Such harmonisation will be crucial to strengthening evidence‐informed integration strategies.

## 4 Conclusion

The central argument of this paper is that TCIM integration is best understood not as a narrow question of clinical efficacy but as a multi‐domain governance challenge, in which evidence, regulation, commerce, communication, and Indigenous authority are tightly coupled. Framed this way, the task is not simply to generate “more trials” but to build plural, fit‐for‐purpose evidence frameworks; not to consult communities but to enable Indigenous leadership; and not to impose a single integration model but to design a spectrum of arrangements that, done well, can itself generate the conditions for better research and stronger accountability. Advancing the WHO Global Traditional Medicine Strategy on these terms will require coordinated action across governments, research institutions, regulatory bodies, and Indigenous communities. The analysis presented here suggests that four priorities deserve particular urgency.

First, any TCIM integration strategy must build on the extensive research into why people use traditional medicine and examine how people actually navigate plural systems of care, rather than how health systems assume they should seek care. Motivations and care pathways vary by context and may reflect limited access to biomedical services, especially in rural areas; mistrust arising from poor‐quality or disrespectful care; cultural understandings of health; and financial barriers. Building trust through community advocates, respectful biomedical care, and culturally responsive delivery is essential both to strengthen existing services and to enable informed, equitable TCIM integration.

Second, robust evidence systems must underpin decision‐making. Without credible data on safety, efficacy, and cost‐effectiveness, generated through local research capacity, shared standards, transparent data, and reliable funding, there is no sound basis for deciding what to integrate, regulate, or reimburse. This requires investment not only in trials but also in pharmacovigilance, economic evaluation and implementation research suited to TCIM's complexity, and agreement on pluralistic frameworks for weighing the heterogeneous evidence such research produces.

Third, expanding regulatory capacity is crucial, especially in countries with the weakest systems and the largest burden of unregulated practice. Regional harmonisation efforts, such as the AMRH programme, offer promise but require stronger political commitment and financing. Because TCIM markets increasingly operate across borders, including via e‐commerce, regulatory cooperation must extend well beyond national jurisdictions.

Fourth, fair and culturally respectful partnerships with Indigenous peoples must be treated as a governance priority. Access and benefit‐sharing frameworks, community‐led oversight, and principles of Indigenous intellectual property sovereignty should guide all stages of research, regulation, and commercialisation, with Indigenous leadership exercised not only over benefit‐sharing but over the design and validation of the evidence itself.

The WHO is uniquely positioned to support these efforts by facilitating global learning, strengthening benchmarking tools, and convening diverse stakeholders to address persistent gaps in accountability, market regulation, and digital misinformation. TCIM will strengthen global health only when evidence, ethics, and equity are not merely aspirational principles, but are embedded in the institutional governance of how traditional medicine is researched, regulated, financed, and delivered.

## Funding

This research was funded by the NIHR Global Health Research Centre for Non‐communicable Disease Control in West Africa (STOP‐NCD) (Grant Reference: NIHR203246) using UK aid from the UK Government to support global health research. The views expressed in this publication are those of the author(s) and not necessarily those of the NIHR or the UK government.

## Ethics Statement

The authors have nothing to report.

## Conflicts of Interest

The authors declare no conflicts of interest.

## Data Availability

Data sharing not applicable to this article as no datasets were generated or analysed during the current study.
